# A Review of Bryophytes: Enzymatic and Non-Enzymatic Antioxidants as a Key for Their Pharmacological Potential and Green-Biotechnological Application

**DOI:** 10.3390/antiox15010016

**Published:** 2025-12-22

**Authors:** Stela Ginin, Toncho Dinev, Neli Grozeva, Neli Memdueva, Milena Tzanova

**Affiliations:** Department of Biological Sciences, Faculty of Agriculture, Trakia University, 6000 Stara Zagora, Bulgaria; toncho.dinev@trakia-uni.bg (T.D.); n.grozeva@trakia-uni.bg (N.G.); neli.memdueva.21@trakia-uni.bg (N.M.)

**Keywords:** bryophytes, mosses, specific antioxidants, secondary metabolites, ROS, enzymes, pharmacological potential, bioreactor

## Abstract

Bryophytes, as early land plants, have evolved and developed a wide array of enzymatic and non-enzymatic antioxidant defense mechanisms to cope with oxidative stress. This review explores the intricate biochemical pathways of bryophyte antioxidant defense including their secondary metabolite (SM) systems and protective enzymes such as superoxide dismutase (SOD), catalase (CAT), glutathione transferases (GSTs), glutathione peroxidase (GPx), and glutathione reductase (GR). These metabolic components function through species-specific regulatory mechanisms related to expression way. The pharmacological significance of bryophyte-derived compounds is also highlighted, supported by recent discoveries of numerous bioactive molecules, such as melatonin, cannabinoids, and specific chemical marker compounds. Most current biochemical studies on bryophytes focus on their desiccation tolerance and their utility as pollution indicators; however, another aim of this review is to underscore their broader pharmacological promise. Furthermore, this paper explores the biotechnological applications of bryophytes in drug discovery and the need for bioreactor cultivation of the species used. It also emphasizes the need for further investigation into bryophyte biochemistry and enzymology, particularly their unique enzyme systems, to fully unlock their therapeutic potential.

## 1. Introduction

Approximately 80% of the global population relies on herbal medicine [1], and around 70% of antibacterial and anticancer medications are derived from natural sources [2,3]. The aging processes and numerous diseases are the result of metabolic disorders and oxidative stress. Oxidative stress plays a significant role with a two-way connection to aging and multiple diseases in living organisms. Reactive oxygen and nitrogen species (ROS and RNS), generated as metabolic byproducts, have the potential to damage the building blocks of living organisms, the macromolecules of proteins, lipids, and DNA. To mitigate these harmful effects, organisms have evolved smart antioxidant defense systems that consist of both enzymatic and non-enzymatic components [4]. In plants, particularly non-vascular types, such as bryophytes, the oxidative stress response is crucial for survival under extreme environmental conditions. “Bryophytes” consists of three divisions: liverworts (about 5000 species), hornworts (about 150 spp.) and mosses (about 20,000 spp.) [5]. They are integral to traditional Chinese, Indian, and American medicine, but despite their therapeutical promise, bryophytes remain largely overlooked in mainstream pharmacology and ethnobotany in Europe [6,7]. Bryophytes represent some of the best plant models for antioxidant research. Their resilience to environmental extremes and minimal resource requirements make them excellent models for studying oxidative stress responses. They flourish in diverse ecosystems, even in nutrient-deficient post-technogenical areas [8] and show season-dependent variation in biologically active compounds (BACs) level [9].

Metabolite stress due to low temperatures and desiccation have similar signaling mechanism and physiological effects, but desiccation-tolerant plants are not necessarily tolerant of low temperatures, and vice versa—many low temperature tolerant organisms cannot survive desiccating conditions [10]. Bryophytes are unique—they are sustainable at low and high temperatures, UV radiation, drought, chemical and biological pollution, show resistance to numerous pathogens that typically affect vascular plants [11,12], and at the same time do not require special conditions to grow. Bryophytes lack feeding potential and very seldom attract herbivores [11] since their metabolism is not designed for accumulating nutrients (carbohydrates, lipids, proteins) but explicitly for economical survival. They survive with limited sunlight and minimal water, while simultaneously they are capable of restoring their normal metabolism and photosynthesis levels in a very short time—ranging from a few minutes to 24 h [13].

These facts stimulate human interest in the biochemical capacity and pharmacological potential of bryophytes, their methods for counteracting oxidative stress, the specific secondary metabolites (SM) involved in their defensive biochemical mechanism, and how we may upgrade and apply our understanding of them. Although still not extensively researched, bryophytes are now acknowledged for their unique biochemical and pharmacological properties, providing novel opportunities for drug discovery and therapeutic applications. This review focuses on the enzymatic and non-enzymatic antioxidants and defense mechanisms of bryophytes as the basis for their pharmacological potential and biotechnological applications.

## 2. Materials and Methods

This study was conducted as a literature review focused on the enzymatic mechanisms, antioxidant systems, and pharmacological potential of bryophytes. In addition, data regarding their applications in biotechnology were incorporated.

### 2.1. Data Sources and Search Strategy

A structured literature search was performed using the following international scientific databases: Google Scholar, ScienceDirect, PubMed, Scopus, and Web of Science. The search encompassed peer-reviewed publications from the period of 2000 to 2025, preferably. The following search terms and keyword combinations were used: “bryophytes”, “mosses”, “antioxidants”, “enzymes”, “phytochemical composition”, “pharmacological activity”, “medicinal use”, “biologically active compounds”, “biotechnology”. Boolean operators (AND, OR) were used to optimize search combinations.

Sources were selected for inclusion based on the following criteria:

- The publication is a peer-reviewed scientific article, review, dissertation, or academic report;
- Written in English;
- Contains relevant keywords in the title, abstract, keywords section, or full text;
- Provides information on phytochemical composition, biological activity, or medical/pharmaceutical application of any of the bryophyte species.

### 2.2. Data Extraction and Analysis

The selection process included

- Preliminary screening of titles and abstracts;
- Full-text review of eligible articles. Extraction and tabulation of relevant data, including identified BACs, reported pharmacological activities, and toxicological profiles, where available;
- Comparative analysis of the phytochemical profiles and documented applications.

All data were categorized by species and analyzed to identify commonalities and distinctions in their antioxidant constituents and pharmacological potential. This approach aimed to provide a synthesized overview of the current knowledge and to identify promising candidates for further exploration in pharmacology, applied phytotherapy, and biotechnology.

### 2.3. Enzyme Classification

Enzyme numbers are listed in the ENZYME nomenclature database, available online [14].

### 2.4. Chemical Structural Drawing

The chemical structures are created using the features of ChemDraw sofware, ver. 23.1.1 (Revvity Signals Software Inc., Waltham, MA, USA).

## 3. Antioxidant Defense Systems of Bryophytes

### 3.1. Classification and Mechanism

**Figure 1:**
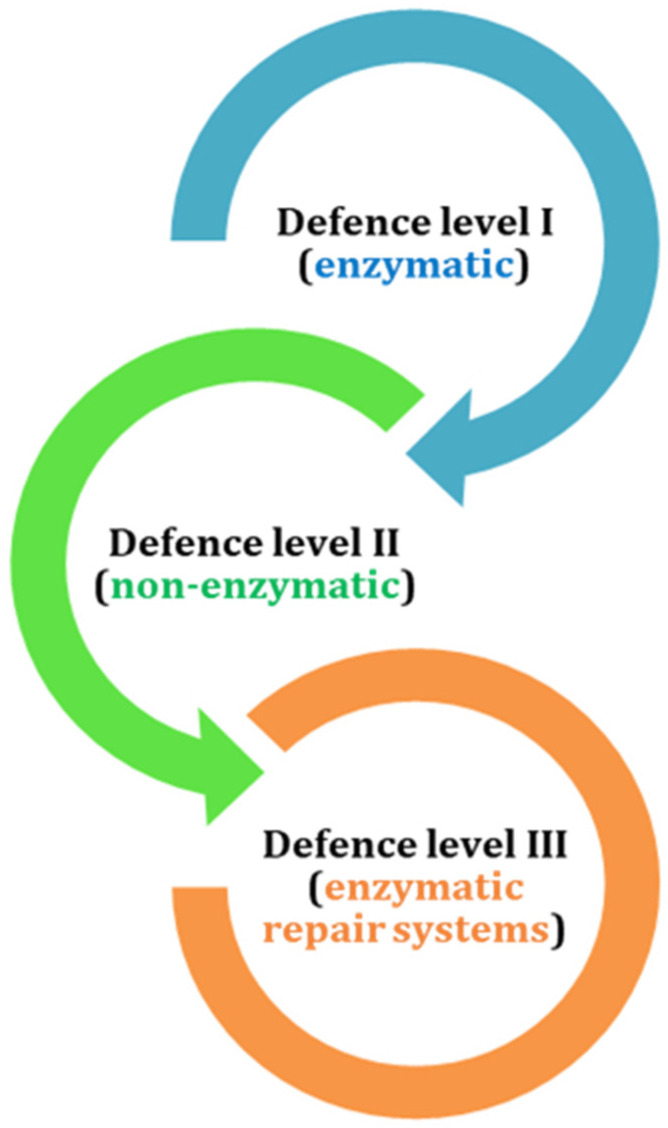
Antioxidant levels of defense of bryophytes (the arrows follow the hierarchy of sequence of action).

The antioxidant defense in living organisms is typically divided into three levels:

- Primary enzymatic defenses (e.g., SOD, CAT, GPx)—the most powerful;
- Secondary defenses from dietary or endogenous non-enzymatic antioxidants (e.g., vitamins A, C, E, flavonoids, GSH);
- Enzymatic repair systems that fix oxidative damage (Figure 1).

In plants, these systems are complemented by specialized adaptations such as suppression of photosynthesis (respectively growth and development), alternative metabolic pathways, e.g., C4- or crassulacean acid metabolism (CAM), and SMs production. Bryophytes are C3 plants [15], indicating that they rely on the rest of the alternative adaptation mechanisms.

### 3.2. Enzymatic Antioxidants of Bryophytes

Antioxidant enzymes are admittedly a main part of the defense mechanism that is present in the majority of plants. These enzymes are favored for biosystematics study over the other enzyme systems due to their important role in physiological processes and abundant list of allozymes. Usually zymogram patterns (activity picture) of peroxidases (PXs) and SODs are utilized to demonstrate the close relationship between mosses and to monitor variations in gene expression resulting from stress conditions [16]. Bryophytes possess a large variety of antioxidant enzymes. Some of them have been investigated and their specific expression has been demonstrated. They can be conditionally categorized into two groups (Table 1).

### 3.3. Common Antioxidant Enzymes of Bryophytes

SOD, PX, and CAT are antioxidant enzymes that vary in the ROS they target, as well as in their mechanisms and products. SOD initiates the process by removing superoxide, while CAT handles large bursts of H_2_O_2_, especially in high-H_2_O_2_ environments. Peroxidases such as GPx operate more precisely, handling low levels of H_2_O_2_ and lipid damage with assistance from glutathione.

Analysis of antioxidant enzymes in *H. propagulifera* reveals a distinct relationship between desiccation and the activities of CAT, SOD, PX, and GR—increasing during desiccation and quickly returning to baseline levels upon rehydration. It is clearly stated that during the desiccation stress, four enzymes are overexpressed to overcome the induced oxidative stress and to unlock defense against the ROS produced. This approach is also working against additional external barriers [19].

Another study involving *Atrichum undulatum* (Hedw.) P.Beauv and *B. rutabulum* was conducted in relation to nitrogen metabolism. Investigations of NR activity in the green tissues of the two moss species were performed in light of its crucial role in nitrogen fixation, which serves as a key growth factor for plants and is linked to their environmental pollution dependency. Based on their results, the authors suggest that mosses NR may be significantly different from that found in algae and higher plants [17]. The other scientific team [18] worked on in silico characterization of NR gene family and on prediction of protein content of the moss *P. patens*, and their results support the suggestion that bryophytes and vascular plants utilize different mechanisms to regulate NR activity.

Glutathione transferase, glutathione S-transferase or GST, is a group of enzymes that are crucial for cellular detoxification. These enzymes catalyze the conjugation of glutathione to various harmful compounds, enhancing their water solubility and simplifying the body process of elimination. GSTs serve as markers of oxidative stress. They catalyze the conjugation of glutathione to xenobiotic substrates [36]. GSTs exhibit structural conservatism while displaying a wide range of functions. They also serve a non-catalytic role by binding flavonoids from the cytosol and contributing to their deposition in the vacuole. They also play a role in UV-triggered signaling pathways and contribute to the regulation of apoptosis [37].

In 1981, Dhindsa and Matowe examined the activity of SOD and CAT during drought in two moss species—*C. filicinum* and *T. ruralis* [21]—and subsequently, in 1991, expanded their research to include glutathione metabolism of *T. ruralis*. They found a positive correlation between GSSG levels and stress conditions and observed a change in the level of GR, GPx, and GTs during different conditions of drought and rehydration and how this affects the protein synthesis. An intriguing discovery is that the activity of these enzymes depends on the rate of the drying and rehydration, and the study showed the activity ranking of the three enzymes: GR > GPx > GTs [20]. The other investigation was on the levels of PX and SOD in *B. caespiticium* as a response to oxidative stress under elevated temperatures and varying light intensity. It revealed altered gene expression due to hyperthermia, which leads to better thermal stability of the produced enzymes [8].

Yuqing et al. examined the enzyme activity of SOD, CAT, and PX in the metabolism of *B. argenteum* as a response to oxidative stress after desiccation, revealing that gradual dehydration results in a stronger stress response, with larger increases in antioxidant enzyme activity and stress-related transcripts compared to rapid dehydration. The study highlights *B. argenteum*’s ability to adapt to harsh environments by modulating its physiological, biochemical, and molecular responses based on the rate of water loss [22].

Another scientific work found elevated PX activity in *D. scoparium*—twice as high as in other mosses in this specific research (*Hylocomium splendens* (Hedw.) Schimp. and *Pleurozium schreberi* (Willd. ex Brid.) Mitt.)*. D. scoparium* maintains a constant high level of PX, which is additionally increased during desiccation stress. Subsequently, it was discovered that some anionic isoforms displayed both pro- and anti-oxidative activities, i.e., peroxidases can generate and detoxify ROSs, leading to the hypothesis that this trait is an evolutionarily ancient characteristic important for plant stress tolerance [23]. Later, in 2021, the same authors added scientific findings about ascorbate peroxidase (APX) and its contribution to stress metabolism [38]. Rzepka [39] observed a reverse suppression effect on the activity of CAT and SOD in *Mnium undulatum* (*P. undulatum*) under stress from a lack of oxygen (hypoxia).

A study examines the alteration in the activity of key enzymes-SOD, PX, and CAT of *F. hygrometrica* and *Amblystegium serpens* (Hedw.) Schimp. antioxidative defense mechanism as a protective reaction against elevated concentrations of copper and zinc [40]. The enzyme pathway of antioxidant defense is a focal point for investigation, especially the activity of GPx and GR in different mosses. The literature sources regarding the content of GSH/GSSG in mosses are rather scarce [41].

The activity of the antioxidant enzymes APX, CAT, and guaiacol peroxidase in *Syntrichia ruralis* (Hedw.) was studied in both the rehydrated and desiccated conditions [42]. During investigation of enzyme content of 10 bryophytes from India, the activity assay of peroxidase, CAT and polyphenol oxidase was carried out using different specific biochemical methods [43].The study revealed an accumulation of reactive oxygen species and a defense against drought stress indicating the activation of drought tolerance mechanisms in this moss species.

A 2017 study by an Anatolian team determined the levels of GSH and GSSG in two moss species from the *Pottiacea* family (*Syntrichia montana* Nees. and *S. ruralis*) by HPLC method, and both species were identified as good sources of glutathione. The experimental findings reveal that both of these bryophytes are promising plants with potent antioxidant effects [41]. Two years later, the team expanded this investigation by including additional moss species (*B. argenteum*, *Imbribryum mildeanum* (Jur.) J.R.Spence, *Ptychostomum imbricatulum* (Müll. Hal.) Holyoak & N.Pedersen, *P. moravicum* (Podp.) Ros & Mazimpaka, and *P. capillare*. The new results indicated that these bryophyte species, belonging to the *Bryaceae* family, are very good sources of glutathione (reduced-GSH and oxidized-GSSG) [44]. The authors concluded that these species are rich in antioxidants, making them suitable raw materials for various applications

Parameters related to oxidative stress, studied in *A. undulatum* through exposure to varying concentrations of cesium acetate at different pH levels (3, 4, 6, 8), showed the activity of antioxidant enzymes (SOD, PX, CAT) and other associated parameters (malondialdehyde content, H_2_O_2_ and total phenolic content) which confirms SOD as the first line of defense against ROS, while PX and CAT are subsequently activated [45]. PX-divergent forms are responsible for UV-stress protection [46]. Mosses (such as *P. patens*) possess several unique or divergent peroxidases, reflecting their early evolution as terrestrial plants and the adaptation to extreme environmental conditions.

Meyer and Angerman studied PAL in *F. hygrometrica*, which is the initial enzyme in the phenylpropanoid pathway responsible for the production of lignins and flavonoids [24]. Its activity is often influenced by biotic and abiotic conditions, and thus it has an indirect but essential role in plant’s defense mechanism against stressors. In 2012, Ponce De León et al. shared findings from their investigation on *P. patens* response to a fungal pathogen, noting that one of the observed effects is the expression of the PAL gene [47].

### 3.4. Specific Antioxidant Enzymes of Bryophytes

Several enzymes examined via specific model moss genomes (*P. patens* or *P. nutans*) have been found and identified, as denoted by the inclusion of the initials “Pp” or “Pn” in their names.

Such cases involve carotenoid cleavage dioxygenases from *P. patens*, named PpCCD7 and PpCCD8. They are responsible for hormone-like signaling and generate SL products [27]. They vary based on substrate: PpCCD7 is responsible for 9-cis-β-carotene oxidation, while PpCCD8 oxidizes either 9-cis-10′-apo-beta-carotenal or all-trans-10′-apo-beta-carotenal. These enzymes are homologs, whose dioxygenase reaction initiates SL biosynthesis—an ancient signaling pathway found across terrestrial plants. In mosses, these two steps replicate the basic biosynthetic conversion of carotenoids to SL—even though *P. patens* lacks the MAX1 P450 enzyme found in flowering plants. SLs in mosses serve unconventional roles: instead of regulating shoot branching (as in vascular plants), they act as quorum-sensing-like signals controlling protonema branching, colony extension, and fungal resistance [48]. This study proposed the hypothesis that the SLs of *P. patens* resemble sensing molecules responsible for communication.

Another particular enzyme activity expression is Mn-SOD, which was found in the moss *B. unguiculata* [25]. The SOD is a glycoprotein. The mitochondrial Mn-SOD remains preserved through evolution and functions as anticipated, while stress-regulated expression patterns further highlight its importance in moss physiology. Mn-SOD in mosses is extracellular germin-like Mn-SOD. It is a notable exception among other plants as it is not a standard mitochondrial SOD but a germin-like protein possessing Mn-SOD activity, further demonstrating the uniqueness of moss metabolism.

AOXs (isoforms) exhibit moss-specific expression profiles linked to oxidative stress protection [26]. AOXs are mitochondrial enzymes which provide an alternative respiratory pathway, bypassing complexes III and IV and reducing reactive oxygen species under stress [49]. AOX itself is not unique to mosses—it has been preserved throughout plant evolution. However, the functional characterization of PpAOX, its impact on moss stress tolerance and cross-organelle redox balance, provides specific insights into moss physiology and adaptation.

*P. patens* ent-kaurene oxidase (PpKO), *P. patens* hydroperoxide lyase (PpHPL), and *P. patens* alternative oxidase (PpAOX) suggest evolutionary divergence from vascular plants [26,34]. Glutathione-related enzyme systems also exhibit considerable diversity and activity during stress conditions, including desiccation and metal exposure. Moss specificity is related to the functional role of ent-kaurenoic acid as a signaling molecule rather than a gallic acid (GA) precursor [26]. The gene responsible for moss-specific cytochrome P450 (ent-KO) associated with terpene biosynthesis, not present in vascular plants, has been identified in *P. patens*. Ent-KO converts ent-kaurene to ent-kaurenoic acid through a three-step oxidation process, which does not proceed to GA as seen in the flowering plants.

Δ6-desaturase is responsible for fatty acid modification, and it is absent in most terrestrial plants [50]. Δ^6^-desaturases found in mosses are key enzymes enabling the synthesis of very long-chain polyunsaturated fatty acids (PUFAs), including arachidonic acid (20:4) and eicosapentaenoic acid (20:5), which are rare in higher plants [29,51]. Mosses accumulate surprisingly high levels of C20 PUFAs (e.g., up to 30% of total fatty acids in *P. patens or Mnium cuspidatum* Hedw.), which are processed into oxylipins—signaling and defensive metabolites—and contribute to their survival in extreme habitats [28].

*P. nutans* flavonoid synthases (PnFNSs) [30] are involved in unique pathways and UV protection mechanisms: they support the transformation of flavanones to flavones. These enzymes are classified into two classes: FNS I and FNS II. In 2020, Wang et al. accomplished the first isolation and recombination of FNS from *P. nutans*. They investigated the potential of recombinant-synthesized enzyme PnFNS I to enhance resilience of other plants (*A. thaliana*) to UV-B radiation, drought, as well as the associated accumulation of ROSs. They reported an increase in flavonoids and improved resistance. This achievement highlights strong potential for future implementation in drug development and therapy.

GSTs (Iota and Hemerythrin) are unique transferases related to detox and ROS defense [31]. GSTs exist in mosses similarly to their presence in vascular plants, bacteria, and animals. In mosses, these enzymes serve critical roles in detoxification and stress response, showing some fascinating differences compared to higher plants. A genome-wide study identified 37 GST genes in the model moss *P. patens*. They belong to 10 different classes along with two moss-specific classes named Hemerythrin and Iota—absent in higher plants [31]. Hemerythrin is a non-heme iron protein that typically transports oxygen. The mechanism of dioxygen binding is unusual. The majority of O_2_ carriers operate via formation of dioxygen complexes, but hemerythrin retains the O_2_ in the form of a hydroperoxide.

Other enzymes—molecular pathway kinases (MPKs)—are part of innate immunity. MPKs play a role in the phosphorylation of proteins in response to fungus pathogens and environmental stressors. They possess a cascade pathway of activity that progresses through several steps of activation by phosphorylation. They are also referred to as stress-activated protein kinases and are known to appear as a pathogen-triggered response in vascular plants, although their activity in nonvascular plants, such as bryophytes, is less understood. An investigation of *P. patens* MPKa and MPKb aimed to clarify their place in moss defense mechanisms [32]. The study hypothesized their specific role in the innate immunity, unlike kinase type enzymes found in vascular plants, which are expressed as an abiotic stress response.

Haq and Kilaru investigation reveals the specific behavior of PpFAAH and its increased activity compared to the same type of enzyme in vascular plants and animals [33]. This enzyme is responsible for hydrolization of fatty acids and especially anandamide.

PpHPL refers to *P. patens* hydroperoxide lyase, an enzyme belonging to the CYP74 family of cytochrome P450-like proteins. It is localized in the inner chloroplast membrane and plays a critical role in the oxylipin pathway, cleaving fatty acid hydroperoxides into volatile aldehydes such as nonenals and oxo-fatty acids. PpHPL isoform is responsible for fatty acid oxidation to various GLVs profiles [34]. These C6 compounds are a crucial component of the defense mechanism of green plants. They are very rapidly synthesized and released in response to herbivore or pathogen attacks and as a reaction to abiotic stress conditions [52]. PpHPL is moss-specific at functional levels, especially in terms of substrate preference for 18 fatty acid hydroperoxides (similar to higher plants) and 20 hydroperoxides which are rare in seed plants but present in mosses. This dual activity is uncommon in seed plant hydroperoxide lyases and points to the unique functional adaptation in mosses. They produce C9-aldehides with defensive and signaling roles by generation of volatile oxylipins upon wounding [53].

ENA-ATPases, which are associated with Na^+^ export at elevated pH levels, are present in mosses and fungi [35] and are absent in flowering plants. Common types of ATPases include the Na, K-ATPase found in animal cells and the H^+^-ATPase present in fungi and plants, and Ca^2+^-ATPase. In fungi, another P-type ATPase, the ENA ATPase, was discovered in 1991; this novel P-type ATPase with yeast origin plays a role in sodium transport [54]. It is found typically in plasma membranes, but it is possible to be present in the inner membranes as well. Initially considered as a solely a fungal enzyme, it is now recognized to exist in bryophytes and protozoa, and it has potential biotechnological applications [55]. It is responsible for salt tolerance and possesses promising potential for novel therapeutic approaches [56].

All these enzymes indicate how bryophytes

- Adapt biochemically to conditions of desiccation, UV light, cold, and low-nutrient environments;
- Produce SMs which are absent in vascular plants;
- Employ different hormonal and signaling pathways (e.g., simplified auxin/cytokinin, modified jasmonate).

This diversity reveals the unique metabolism of bryophytes determined by conditions they must survive. The available sources on exploration efforts in the frame of enzyme systems in bryophytes are rather scarce, and actually, antioxidant enzymatic activities have yet to be investigated to the necessary extent.

### 3.5. Non-Enzymatic Antioxidants of Bryophytes

SMs are not necessarily involved in the main processes of growth and development, but at the same time they are linked to them through strong impacts on emergency conditions. They are related to specific needs–defense, communication, and competition in various biotic and abiotic factors. In the past, many of these compounds were considered as metabolic wastes, and they were named “shunt metabolite” or “idiolite” [3], but they are now acknowledged for their critical roles in plant survival and pharmacological potential.

Bryophytes generate a wide variety of SMs as an antioxidant defensive response, such as flavonoids, terpenes, polyphenols, cannabinoids, oxylipins [57,58], and even melatonin, which belongs to the alkaloid family. Table 2 displays some specific SMs identified in bryophytes and classified according to their molecular composition and structure into several basic chemical groups of compounds.

Regardless of their diversity and taxonomic specificity, there are three general biochemical synthetic pathways: (1) glycolysis is the main source of precursors for biosynthesis of terpenoids, saponins, phenols, flavonoids, polyketides, fatty acids; (2) tricarboxylic acid cycle (Krebs cycle) is a hub for many pathways and thus is responsible for production of variety of SMs including alkaloids; and (3) shikimate way intermediates are involved in synthesis of monoterpenes, alkaloids, flavonoids, and quinones [3]. Usually sources are “shared” or so-called “hybrid” just as shikimate-malonate path or shikimate and polyketide ways which derives some bibenzyls and ohyoensins [3,88,89].

SMs can be found in plants free or linked to other compounds (carbohydrates, organic acids, other active compounds) if there is need to be stored. Presented in Table 2, substances are isolated and identified in bryophytes in their pure form.

#### 3.5.1. Phenols

Specific for bryophytes phenolic compounds is sphagnum acid extracted from *Sphagnum* genera [59]. This compound possesses enzyme-suppresing properties, thereby exerting an inhibitory effect on the microbial decomposition of matter, alongside other specific compounds such as shagnans [90].

Quercetin is a flavonol which belongs to the large phenol family and is well-known for its strong antioxidant activity. Under certain conditions, phenols including flavonoids can function as pro-oxidants: quercetin serves as a pro-oxidant at high iron concentrations [91] and is also found in bryophytes (Table 2). Their synthesis follows the shikimate pathway and contributes to oxidative stress resistance [92]. Ohioensins represents a group of flavonoids detected for the first time in *P. ohioense* and thus named after it [63].

Lignans, as a type of polyphenolic compounds, have demonstrated pharmagologycal activity [93]. They are detected in liverworts and hornworts, but not in mosses, which do not contain the enzymes necessary for their formation [67].

Tannins are a subgroup of the polyphenol family, and although they are considered less typical for bryophytes, there is data on the total tannin content in *B. argenteum* and *Dumortiera hirsuta* (Sw.) Nees [94] that indicate a comparable amount to other BACs. Together with polyketides, they display a wide range of biological activities, often specific to the species or genus.

#### 3.5.2. Isoprenes

In total, over 50,000 terpenes and terpenoids have been identified in plant compounds. Bryophytes encompass interesting bioactive representatives—amyrin, marsupellon, diplophyllin, found in *Grimmia*, *Diplophyllum*, and *Marsupella* spp. (Table 2).

Other key non-enzymatic antioxidants in bryophytes with a polyisoprene (tetraterpene) structure include zeaxanthin. This is a xantophyl from the carotenoid family that plays a protective role against DNA damage caused by increased UVB radiation [73].

The specific steroidal compound detected in *M. polymorpha* and *P. patens* is castasteron [74]. An intriguing representative is marsupellon and its derivatives, which belong to the sesquiterpene group, isolated from *M. emarginata* [72].

#### 3.5.3. Cannabinoids

This compound class is represented by anandamide and perrottetinene (Table 2). The first one is PUFA derivative found in the moss *P. patens* [76]. Its presence indicates biochemical functions not preserved through evolution in higher plants [33]. Perrottetinene is a tetrahydrocannabinol (THC)-structural analog, which is detected in *Radula* spp. [75], and the last years is the object of effect investigation due to this similarity. This structural similarity draws attention to the study of the biochemical effect as well.

#### 3.5.4. Bibenzyls

This is a group of compounds that is characterized by great diversity and is typical of liverworths. Marshantins, riccardins, asterelins, and plagiochyns are a few of the representatives of this category (Table 2).

#### 3.5.5. Alkaloids

A representative of this class is melatonin, which supports stress tolerance through ROS scavenging, abscisic acid interaction, and SMs synthesis [57]. Also, evidence shows that melatonin enhances biosynthesis of other SMs, such as brassinosteroids and jasmonic acid, and even has a positive effect on the activity of antioxidant enzymes, thereby further amplifying ROS resistance [95].

#### 3.5.6. Vitamins

Important studies on bryophyte phytochemicals show the presence of certain vitamins, including riboflavin (vit. B2), which is present in some mosses from *Barbella* family (*B. pendula*, *B. enervis*, *F. nipponica*, *H. plumaeforme*, and *N. nitidula*). *A. undulatum* and *M. hornum* produce vitamin E (α-tocopherol) [85,86]. Vitamin C (L-ascorbic acid) has been found in ethanolic extract of *B. capillare* [81] and has also been determined in high amounts in extract from *H. cupressiforme* [84].

#### 3.5.7. Oxylipins

These compounds are oxidized products of fatty acids (FAs). They can be produced via two distinct metabolic pathways—enzymatic and non-enzymatic. As a result of their biochemistry, some of the primary SMs can be generated, such as the plant hormone jasmonic acid, along with green leaf volatiles (GLVs) as well [96].

## 4. Pharmacological Potential of Bryophytes

Since ancient times, humans have depended on plants not only for providing basic necessities, but also to fill the requirement for medicines [97]. In this respect, as a special plant group, bryophytes produce unique SMs with huge diversity of bioactivities, some of which can serve as chemo markers. Such SMs are listed below:

### 4.1. Phenols

This group includes polyphenols, tannins, flavonoids, and lignans (Table 2), which have anti-inflammatory, anticancer, antioxidant, and cardiovascular healing effects [98,99]. Sphagnum acid and sphagnan (a pectin–like polysaccharide) are isolated from cells of *Spagnum* moss, recognized for its antiseptic and wound healing properties for decades [59,100].

Lignans, represented by jamesopyrone and epiphyllic acid, exhibit diverse activities including antioxidant, anti-inflammatory, antibacterial, antifungal, antiviral, antitumor, antimitotic, neuroprotective, hormonal–due to their estrogen-like properties, cardiovascular support, and metabolic regulation [101].

Ohioensins are a group (A to H) of flavonoid-like compounds isolated form *Polytrichum* species, that show a variety of pharmaceutical properties such as cytotoxic, antioxidant, and protein inhibition [89].

Polyketides, with their huge variety of compounds, indicate numerous pharmacological activities such as antibacterial, antiparasitic, antitumor, cholesterol-lowering, and immune-suppressing effects [102]. The information regarding their content in *P. patens* [103] aligns with the characteristics noted for this species.

Leucobryns represent another specific group of compounds—aromatic pigments extracted from the moss *P. longifolium*—exhibiting potential cytotoxic properties [69].

### 4.2. Isoprenes

Terpenes and terpenoids have antimicrobial, anticancer, and antipyretic effects and metabolism disorder healing activity [104]. Saponins, belonging to this group, possess fungicidal, nematocidal, viricidal, bacteriocidal, and insecticidal activity [105].

Marsupellons are sesquiterpenes derived from liverworth family *Marsupella* that possess cytotoxicity [106]. Diplophyllin, a sesquiterpene lactone isolated from *Diplophylum* sp., expresses the same activity [71].

Castasterone, belonging to the steroid group, is a brassinosteroid plant hormone that has anticancer properties with effects on both drug-sensitive and drug-resistant cells [107].

Carotenoids, such as zeaxantine, are strong free radical scavengers and so known for their antioxidant activity [108]. However, they also possess antimicrobial, anti-inflamatory, antiproliferative, and anti-angiogenic properties [109,110].

### 4.3. Cannabinoids

Perrottetinene exhibits THC-similar painkiller effect without significant psychoactivity [111]. Anandamide is another representative of this group, which possess anticancer and anti-inflammatory properties against endotoxins [112]. This endocannabinoid is mostly related to emotional biochemistry and brain processes in humans, and its discovery as part of the metabolism of *P. patens* is interesting and surprising. It is assumed that the role of anandamide in this moss is as the subject of abiotic stress-responding pathways [76].

### 4.4. Bibenzyls

Marchantin is a bibenzyl isolated from liverworth Marshantia family that demonstrates antifungal and cytotoxic properties and even snake anti-venom effects [113]. Plagiochins are also a group of macrocyclic bis(bibenzyl) compounds isolated from liverworts, particularly from the genera *Plagiochila* and *Marchantia* [81]. These compounds exhibit antifungal properties through chitin synthesis inhibition [82].

### 4.5. Alkaloids

The plant hormone melatonin belongs to the alkaloid group of indols and possesses antifungal properties [114]. There is also evidence of mercury absorption inhibition and enhanced resistance to Hg toxicity in moss species [58].

### 4.6. Vitamins

The benefits of vitamins, particularly C and E, are widely recognized thanks to their synergistic antioxidant properties that safeguard cells from oxidative damage.

### 4.7. Oxylipins

Oxylipins as oxidized products of PUFAs, may serve as biomarkers for oxidative stress response. They also play a role in inflammatory-related diseases, obesity, and diabetes [115]. The plant hormone jasmonic acid exhibits a sedative effect [116] and along with its derivatives possesses also anti-inflammatory, anticancer, and certain cosmetic benefits [117].

### 4.8. Antimicrobial Activity

Flavonoids, terpenoids, polyphenols, bibenzyls, and fatty acids derivatives—a lot of chemical compounds are famous for their antibacterial, antifungal, and cytotoxic effects [118]. One of the main focuses in bryophyte exploration is exactly the antimicrobial effect of their extracts. In this respect, two primary reasons exist: the pursuit of substitutes of conventional antibiotics and other antimicrobial drugs, along with the possible lack of resistance in pathogens to bryophyte preparations, as well as exploring methods to utilize it for human benefit. A typical investigation approach encompasses a test of effectiveness of various bryophyte preparations against different bacteria strains (G+ and G−) and fungi, determination of inhibition zones, minimum inhibitory concentration, minimum bactericidal/fungicidal concentration, and finding the relationship between substance content and activity (SAR) [119,120,121,122,123,124,125], and in addition, determination of environmental conditions which influence its maximum effectiveness [9,126].

Michał Dziwak et al. presented synthesized information about the effectiveness of some bryophytes against bacteria–Gram-positive (*Staphylococcus aureus*, *Enterococcus faecalis*, *Streptococcus* spp., *Bacillus* spp.), Gram-negative (*Agrobacterium tumefaciens*, *Citrobacter diversus*, *Enterobacter* spp., *Erwinia chrysanthemi*, *Escherichia coli*, *Klebsiella pneumoniae*, *Listeria monocytogenes*, *Micrococcus flavus*, *Moraxella catarrhalis*, *Proteus* spp., *Pseudomonas aeruginosa*, *Salmonella* spp., *Shigella* spp., *Xanthomonas phoseoli*), and fungi (*Aerobasidium pullulans*, *Aspergillus* spp., *Botrytis cinerea*, *Candida albicans*, *Cladosporium cucumerinum*, *Fusarium oxysporum*, *Penicillium* spp., *Phythophthora infestans*, *Pichia* spp., *Pyricularia oryzae*, *Rhizoctonia solani*, *Saccharomyces cerevisiae*, *Sclerotium rolfsii*, *Septoria tritici*, *Tilletia indica*, *Trichoderma viride*, *Trichophyton mentagrophytes*, *Zygosaccharomyces bailii*). That work found the noteworthy trend that the Gram-negative bacteria are more susceptible to bryophyte preparations compared to Gram-positive ones, which is not typical for the vascular plants [118]. Also, a prominent trend in all antimicrobial studies is the more frequent application of in vitro research methods compared to in vivo, with authors commonly employing well or disc diffusion and serial dilution methods, demonstrating the greater effectiveness of polar solvent extracts (water, methanol, ethanol, acetone, ethyl acetate) [118,121,127].

Based on several ranking criteria (placing the greatest emphasis on drug resistance) the World Health Organization (WHO) has classified the most dangerous for humans as bacteria [128] and fungi [129]. Table 3 shows encouraging positive results regarding the activity of bryophytes against these pathogens.

### 4.9. Other Biological Activities

Numerous researchers also focus on cytotoxicity, which has promising potential for anti-HIV and cancer treatment of leukemia, melanoma, glioma, hepatoma, and breast, ovarian, prostate, and lung cancer [77,113,118,119,120,157].

While current findings are encouraging, additional research is required. The active compounds responsible for the observed effects need to be isolated, identified, and thoroughly tested including other unexplored or poorly explored high-priority pathogens (such as *Shigella* spp. from bacteria and *Candida auris* from fungi). Studies should additionally focus on the particular mechanisms of action and potential applications in drug development or identifying synergistic effects which could be a possible way for reducing synthetic antibiotic dosages.

## 5. Biotechnological Potential of Bryophytes

*P. patens* is the first non-seed plant to have its genome sequenced [158]. Biotechnologically, bryophites such as *P. patens* are utilized in molecular farming to produce complex therapeutic proteins, including recombinant human Factor H and α-galactosidase [158,159]. A significant achievement is the first approved moss-derived medication: Moss-aGal (α-galactosidase for Fabry disease) which passed a successful phase I trial [160]. The latest information relates to promising results after testing according to various criteria: formulation, route of administration, distribution in tissues, localization in organs, and efficacy [161]. Additional studies will determine the optimal dosage and application schedule.

Another accomplishment is related to development of therapeutics through recombinant genes associated with the genome of *P. patens* and involved in

- Expression of taxadiene synthase gene from *Taxus brevifolia* in *P. patens* to produce precursors for the anticancer diterpene, paclitaxel (Taxol™ is utilized for breast, ovarian, and lung cancer treatment) [162].
- The alternative medication for malaria treatment based on artemisinin (a sesquiterpene lactone) was produced through bioengineering of five artemisinin biosynthetic pathway genes into *P. patens*, achieving remarkable yield close to natural levels found in the original plant *A. annua* within a brief period of three days [163].
- The biotechnological approach is employed for the production of other terpenes from *P. patens*. A number of diterpene synthases (diTPS) enzymes were combined to generate industrially important diterpenes [164] and some sesquiterpenes that are valuable for the perfume industry (patchoulol and β-santalene), achieving high yields in a short duration [165].

Another direction for research related to mosses is leveraging their microbiome (e.g., *Sphagnum*) as a source of industrially interesting enzymes and natural products for biotechnological and biomedical applications [166]. The simultaneous integration within silico models opens a huge horizon for practical scientific–biotechnological relationship. Moss-based bioreactors and in silico predictive models alongside structure-activity relationship (SAR) and quantitative structure-activity relationship (QSAR) approaches [167,168] represent the future in the discovery and creation of novel plant-derived therapeutics.

A disadvantage of utilizing bryophytes as broadly useful biosources is their low concentration levels, which in turn requires a significant plant amount. Another such essential reason arises from their heavy metal accumulation potential [169,170] and the corresponding need to clean the extracts. Thus, bryophyte cultivation in bioreactors is an appropriate method for achieving pure yields under controlled conditions, while conserving biodiversity and protecting the environment. It pertains to options for protection of endangered species as well. Despite their bio-resilience, it is difficult to avoid anthropogenic factors, which is why certain species are classified as “threatened” or “vulnerable”. Acid rains, habitat destruction, urbanization, agricultural practices, and environment pollution result in the first Red List for bryophytes which includes 22.5% of the European species [171].

## 6. Future Research Directions

The scientific research on bryophyte species indicates their large pharmacological potential. From the information provided, we can propose several recommendations regarding the biotechnological and pharmacological applications in the subsequent areas:

- Antioxidant enzymes may play a role in antioxidant therapy, neuroprotection, and anti-inflammatory treatments;
- CATs and PXs can be utilized for their ability to break down hydrogen peroxide into water and oxygen, suitable for anti-aging care and total oxidative stress protection;
- UV-protective enzymes–photolyases can serve as sunscreen ingredients in pharmacology and cosmetics;
- DNA repair enzymes, along with their potential to enhance genome stability under stress, could be utilized in enhancing DNA repair pathways for the treatment of different congenital genetic malformations;
- Antimicrobial enzymes, such as chitinases and β-1,3-glucanases, which break down fungal cell walls, show potential for topical antifungal agents, food preservatives, and in medicine as a possible solution for antimicrobial resistance to conventional antifungal agents;
- Protease inhibitors could regulate inflammation or immune responses and may serve as a possible therapy for autoimmune diseases;
- Secondary metabolism-related enzymes–terpene synthases produce bioactive terpenoids recognized for their antimicrobial and anticancer activity, making them potential drug precursors;
- Polyphenol oxidases exhibiting antioxidant effects hold promise as natural therapeutic sources of antioxidants.

Nonetheless, comprehensive research on their complete pharmacological capabilities is rather scarce. Future research should prioritize the clinical validation of isolated bioactive compounds to establish their efficacy and safety for therapeutic applications. Additionally, further studies should explore the mechanisms of action, pharmacokinetics, and potential synergistic effects between different compounds. We propose future deeper research directions with a focus on the subsequent subjects:

- Quantitative analysis of enzyme activity profiles, which will disclose the metabolic status and specific potential of species;
- Analysis of the huge seasonal and species-specific variation in antioxidant activity;
- Revealing additional chemotaxonomic markers for bryophyte genera and families, facilitating more accurate and reliable affiliation identification, thus alleviating one of the limitation factors for bryophyte investigation;
- Application of QSAR and SAR models for SMs research as a contemporary approach for exploration that avoids the extraction difficulties, the limitations of low content, and the requirements of a huge sample amount;
- Merging biotechnology (bryotechnology) with in silico pharmacology to discover new BACs and facilitate eco-friendly drug production, reducing cost- and time-consuming in vitro and in vivo testing;
- Gene banking that serves as a method to conserve “at risk”species and their cultivation.

**Figure 2:**
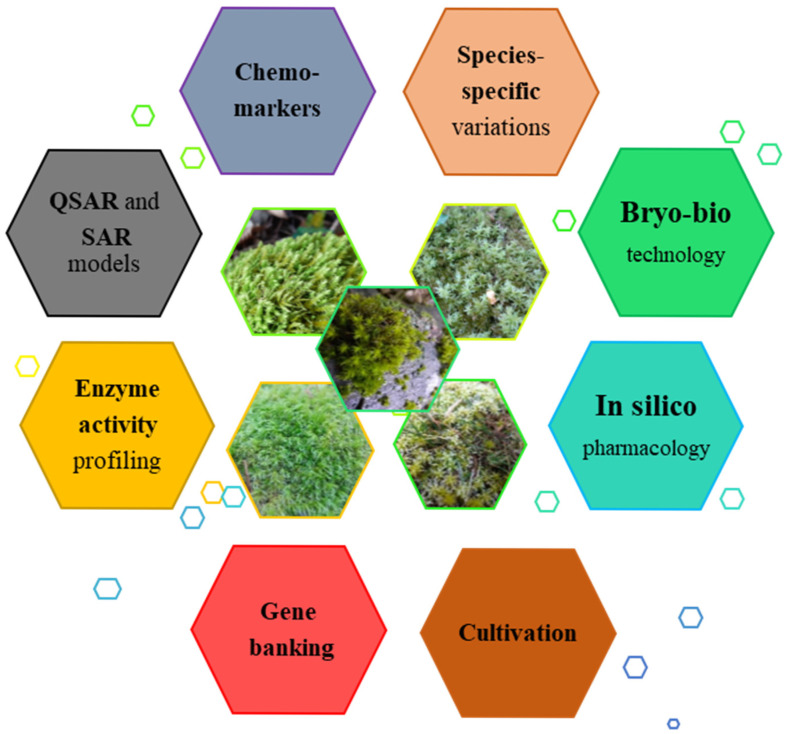
Deeper research direction needs.

Our proposals are illustrated in Figure 2:

## 7. Conclusions

Through systematizing the knowledge regarding the antioxidant defense mechanisms of bryophytes, this study clarifies how these organisms are biochemically adapted to extreme biotic and abiotic conditions and highlights the production of specific SMs, which are absent in vascular plants. This review confirms the uniqueness of bryophyte metabolism, emphasizing their distinct hormonal and signaling pathways aligned with the environment they must survive. Their surprisingly rich biochemical content is a source of BACs (flavonoids, terpenes and terpenoids, phenols, cannabinoids, phytosterols, bibenzyls, alkaloids, vitamins, saponins, oxylipins, tannins, lignans, and polyketides), recognized for their pharmacological and therapeutic effects (ranging from anti-inflammatory, antimicrobial, and total antioxidant activity to cardio-vascular, anticancer, and epilepsy treatments). In bryophytes, certain active compounds have been identified that were once considered to be exclusive to evolutionary higher plants and animals (such as melatonin and artemisinin). This review also exposes the promising potential of bryophytes as “green biolabs” for active molecules harvesting, bioengineering, and drug development. They are also an indispensable part of ecological balance and serve as a sensitive biochemical marker for climate change and environmental deviations. Gradually increasing attention is being paid to the issue of preserving their diversity. Out of their huge variety (more than 25,000 species), only a tiny fraction has been investigated regarding their composition, functions, and particular metabolic mechanisms, indicating a clear scientific gap in this area and opening a huge horizon for further research.

## Figures and Tables

**Table 1:** Antioxidant enzymes in bryophytes.

Enzyme/EC Number	Activity	Bryophyta Species Investigated	Reference
**Common enzymes**			
NR-nitrate reductase (family; number depends on the electron donor) EC 1.7.1.1-3 (NADPH)	Reduces nitrate to nitrite	*Brachythecium rutabulum* (Hedw.) Schimp., *Plagiomnium undulatum* (Hedw.) T.J.Kop., *Physcomitrella patens* (Hedw.) Mitt.	[17] [18]
GR-glutathione reductase EC 1.8.1.7	Transform oxidized glutathione (GSSG) to its active reduced form (GSH) in presence of hydrogen NADH+	*Hyophila propagulifera* Broth. *Tortula ruralis* (Hedw.) G. Gaertn., B. Mey. & Scherb.	[19] [20]
CAT-catalase EC 1.11.1.6	Decomposition of H_2_O_2_ with the release of singlet oxygen	*H. propagulifera*, *Cratoneuron filicinum* (Hedw.) Spruce, *T. ruralis*	[19] [21]
PXs-peroxidases EC 1.11.1.7	Scavenging ROS	*H. propagulifera*, *Bryum caespiticium* Hedw. *Bryum argenteum* Hedw. *Dicranum scoparium* Hedw.	[19] [8] [22] [23]
GPx-glutathione peroxidase EC 1.11.1.9	Catalyze reduction (e.g., scavenging of peroxides)	*T. ruralis*	[20]
SOD–superoxide dismutase EC 1.15.1.1	Convert superoxides to H_2_O_2_ and O_2_	*H. propagulifera*, *C. filicinum*; *T. ruralis*	[19] [21]
GST-glutathione transferase EC 2.5.1.18	Scavenge xenobiotics	*T. ruralis*	[20]
PAL-phenylalanine ammonia lyase EC 4.3.1.5	Initiats the phenylpro-panoid pathway in the synthesis of lignins and flavonoids	*Funaria hygrometrica* Hedw.	[24]
**Specific enzymes**			
Mn-SOD	ROS scavenger and stress response	*Barbula unguiculata* Hedw., *Marchantia paleacea* Bertol.	[25]
AOX-alternative oxidase EC 1.10.3.11	Stress tolerance and cross-organelle redox balance	*P. patens*	[26]
Carotenoid cleavage dioxygenases CCD7 EC 1.13.11.68 CCD8 EC 1.13.11.69/70 * acc. substrate	initiates strigolactone (SL) biosynthesis	*P. patens*	[27]
KO-kaurene oxidase (ent-kaurene oxidase) EC 1.14.13.78	Involved in terpene biosynthesis	*P. patens*	[26]
Δ^6^-desaturases EC 1.14.19.3	Involved in fatty acid modification	*P. patens*, *Ceratodon purpureus* (Hedw.) Brid.,	[28] [29]
FNS-flavon synthase EC 1.14.20.5	Involved in flavone synthesis	*Pohlia nutans* (Hedw.) Lindb.	[30]
GST–iota GST-Hemerythrin EC 2.5.1.18	Involved in ROS defense and detox	*P. patens*	[31]
MPK-molecular pathway kinase EC 2.7.11.24	Involved in fungal pathogen response	*P. patens*	[32]
FAAH-fatty acid amide hydrolase EC 3.5.1.99	Hydrolyzes anandamide	*P. patens*	[33]
HPL-hydroperoxide lyase EC 4.2.99 *	fatty acid oxidation to green leaf volatiles (GLVs)	*P. patens*	[34]
ENA-ATPase– exitus Na-type adenosine triphosphatase EC 7.2.2.13	Related to Na^+^ export at high pH- salt tolerance	*P. patens*, *Marchantia polymorpha* L.	[35]

* Depending on the substrate.

**Table 2:** Specific secondary metabolites and their presence in bryophytes.

Compound Class/ Subclass	Specific Representative	Bryophyte spp.	Reference
**Phenols**			
Phenolic acids	Sphagnum acid 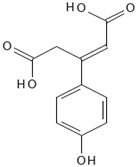	*Sphagnum* spp.	[59]
	Salicylic acid 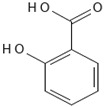	*Sphagnum* spp.	[59]
Flavonoids	Quercetin 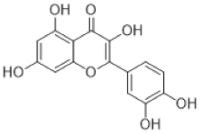	*Lunularia cruciate* (L.) Dumort. ex Lindb., *Philonotis revoluta* Bosch & Sande Lac., *Hypnum cupresiforme* Hedw.	[60] [61] [62]
	Ohioensins (Ohioensin A) 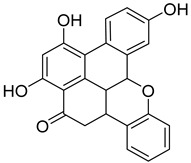	*Polytrichum ohioense* (Ren. & Card.) G.L. Sm., *Polytrichum commune* Hedw., *Polytrichastrum alpinum* (Hedw.) G.L.Sm.	[63,64] [65] [66]
Lignans	Epiphyllic acid 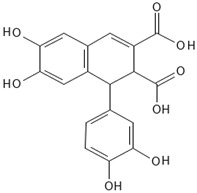	*Calypogeia azurea* Stotler & Crotz, *Lophocolea heterophylla* (Schrad.) Dumort., *Aneura pinguis* (L.) Dumort., *Haplomitrium mnioides* (Lindb.) R.M.Schust, *Jamesoniella autumnalis* DC., *Marsupella emarginata* (Ehrh.) Dumort.	[67,68]
	Jamesopyrone 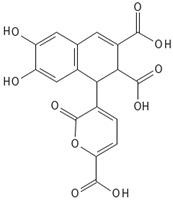	*J. autumnalis*, *M. emarginata*, *Bazzania trilobata* (L.) Gray	
Aromatic pigments	Leucobryns (Leucobryn A) 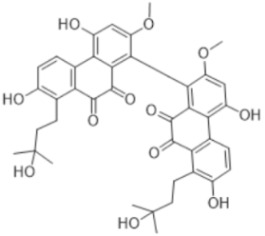	*Paraleucobryum longifolium* Hedw.	[69]
**Isoprenes**			
Terpenes	α-Amyrin 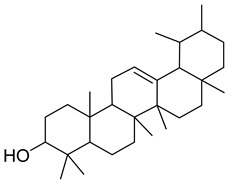	*Grimmia* spp.	[70]
	Diplophyllin 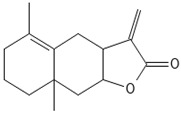	*Diplophyllum* spp.	[71]
	Marsupellon 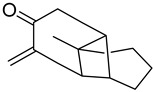	*M. emarginata*	[72]
Carotenoids	Zeaxanthin 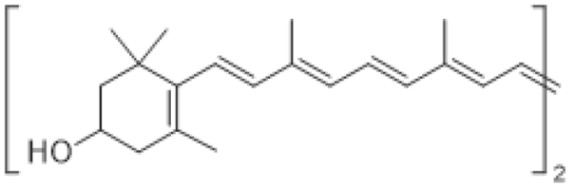	*Bryum pseudotriquetrum* (Hedw.) G.Gaertn., B.Mey. & Scherb., *C. purpureus*, *Schistidium antarctici* (Cardot) L. Savic. & Smirn	[73]
Steroids	Castasteron (brassinosteroid) 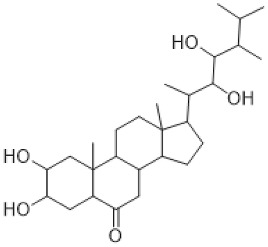	*M. polymorpha*, *P. patens*	[74]
**Cannabinoids**			
Cannabinoids	Perrottetinene 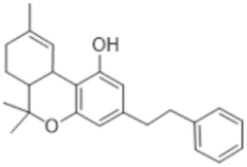	*Radula perrottetii* Gottsche, *Radula marginata* (Hook.f. & Taylor) Gottsche, Lindenb. & Nees and other *Radula* spp.	[75]
Endocanabinoids	Anandamide 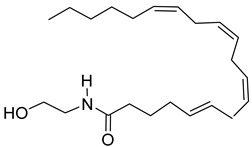	*P. patens*	[76]
**Bibenzyls**			
Bibenzyls	Marchantins Marchantin A 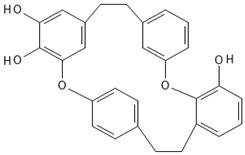	*M. polymorpha*	[77,78]
	Riccardins (Riccardin A) 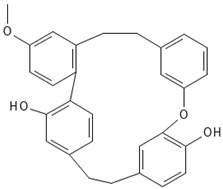	*Riccardia multifida* (L.) Gray	[77,79]
	Asterelins (Asterelin A) 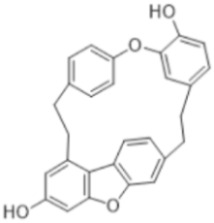	*Asterella angusta* (Steph.) Pandé, K.P.Srivast. & Sultan Khan	[80]
	Plagiochins (Plagiochin E) 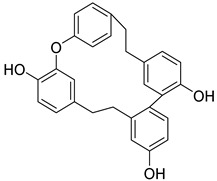	*M. polymorha* *Plagiochasm intermedium* Lindenberg. & Gottsche	[81] [82]
**Alkaloids**			
Indols	Melatonin 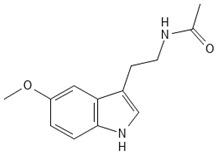	*Taxiphyllum taxirameum* (Mitt.) M.Fleisch	[58]
**Vitamins**			
Water-soluble	Vitamin C (L-ascorbic acid) 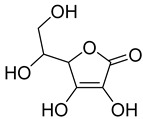	*Bryum capillare* Hedw., *H. cupressiforme*	[83] [84]
	Vitamin B2 (Riboflavin) 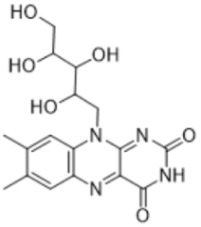	*Barbella pendula* (Sull.) M. Fleisch, *Braunfelsia enervis* (Dozy & Molk.) Paris, *Floribundaria nipponica* Nog., *Hypnum plumaeforme* Wilson, *Neckeropsis nitidula* (Mitt.) M.Fleisch.	[85,86]
Fat-solible	Vitamin E- (α-tocopherol) 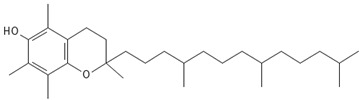	*A. undulatum*; *Mnium hornum* Hedw., *Radula* sp.	[75,85,86]
**Oxylipins**			
Plant hormone	Jasmonic acid 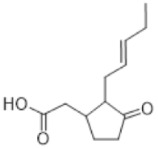	*Anthoceros agrestis* Paton, *A. punctatus* L., *Phaeoceros laevis* (L.) Prosk.; *F. hygrometrica*, *Polytrichum juniperinum* Hedw., *Hedwigia ciliata* (Hedw.) P.Beauv., *Conocephalum conicum* (L.) Dumort, *M. polymorpha*	[87]

**Table 3:** Antimicrobial activity of bryophyte species against bacteria (critical and high priority group, [128]) and fungi (critical priority group, [129]).

Susceptible Microbial Species	Active Bryophyte Species	References
**Bacteria**		
*Klebsiella pneumoniae* (G−)	*Dryptodon pulvinatus* (Hedw.) Brid., *D. scoparium*, *B. argenteum*, *Schistidium crassipilum* H.H.Blom, *Orthotrichum anomalum* Hedw.	[130]
	*P. undulatum*	[130,131]
	*Brachythecium glareosum* (Bruch ex Spruce) Schimp., *Scleropodium touretii* (Brid.) L.F.Koch, *H. cupressiforme*	[132]
	*Taxithelium nepalense* (Schwägr) Broth	[133]
	*H. cupressiforme*, *Homalothecium sericeum* (Hedw.) Schimp., *H. lutescens* (Hedw.) H.Rob., *Ctenidium* molluscum (Hedw.) Mitt., *Thuidium delicatulum* (Hedw.) Schimp., *Leucodon sciuroides* (Hedw.) Schwägr., *Eurhynchium striatulum* (Spruce) M.Fleisch.	[134]
	*B. argenteum*, *Plagiochasma appendiculatum* Lehm. & Lindenb., *Mnium marginatum* (Dicks.) P.Beauv., *C. conicum*	[135]
*Escherichia coli* (G−)	*D. pulvinatus*, *B. argenteum*, *S. crassipilum*, *O. anomalum*	[130]
	*P. undulatum*	[130,131]
	*H. cupressiforme*, *H. sericeum*	[131]
	*D. scoparium*	[130,136]
	*B. glareosum*, *S. touretii*, *H. cupressiforme*	[132]
	*Sphagnum magellanicum* Brid.	[137]
	*T. nepalense*	[133]
	*Homalothecium nitens* (Hedw.) H.Rob., *C. molluscum*, *E. striatulum*, *H. cupressiforme*, *H. sericeum*, *T. delicatulum*, *H. lutescens*, *L. sciuroides*	[134]
	*B. argenteum*	[138]
	*A. undulatum*	[126]
	*B. argenteum*, *P. appendiculatum*, *M. marginatum*	[135]
	*Pallavicinia lyellii* (Hook.) Carruth.	[139]
	*Jungermannia exsertifolia* subsp. *Cordifolia* (Dumort.) Vána	[140]
*Shigella sonnei* (G−)	*T. nepalense*	[133]
	*H. sericeum*, *L. sciuroides*, *C. molluscum*, *T. delicatulum*, *H. lutescens*, *E. striatulum*	[134]
*Pseudomonas aeruginosa* (G−)	*D. scoparium*	[141]
	*B. glareosum*, *S. touretii*, *H. cupressiforme*	[132]
	*T. delicatulum*, *C. molluscum*, *H. sericeum*, *H. lutescens*, *L. sciuroides*, *E. striatulum*, *H. cupressiforme*	[134]
	*P. commune*	[142]
	*Tortella tortuosa* (Hedw.) Limpr.	[143]
	*H. sericeum*	[144]
	*Leptodictum riparium* (Hedw.) Warnst.	[145]
	*Grimmia pulvinata* (Hedw.) Sm., *Tortula subulata* Hedw., *Weisia controversa* Hedw., *L. sciuroides*, *H. cupressiforme*, *H. sericeum*, *Neckera complanata* (Hedw.) Huebener, *M. undulatum*	[131]
	*H. splendens*, *P. schreberi*	[119]
	*A. undulatum*	[126]
	*B. argenteum*, *P. appendiculatum*, *M. marginatum*, *C. conicum*	[135]
*Salmonella typhi* (G−)	*S. magellanicum*	[137]
	*D. hirsuta*	[146]
	*A. undulatum*	[147]
*Mycobacterium tuberculosis* (G+)	*Thuidium recognitum* (Hedw.) Lindb., *Leucobryum glaucum *(Hedw.) Ångstr.	[148]
*Enterococcus faecium* (G+)	*P. patens*	[149]
	*C. purpureus*	[150]
*S. aureus* (G+)	*P. undulatum*	[120,130]
	*Plagiomnium cuspidatum* (Hedw.) T.J.Kop.	[120]
	*Neckera crispa* Hedw., *Platyhypnidium riparioides* (Hedw.) Dixon, *Abietinella abietina* (Hedw.) M.Fleisch., *C. filicinum*, *Campylium protensum* (Brid.) Kindb.	[151]
	*A. undulatum*	[126,152]
	*D. scoparium*	[152]
	*B. argenteum*	[130,138]
	*B. argenteum*, *P. appendiculatum*, *M. marginatum*	[135]
	*C. purpureus*, *Bartramia pomiformis* Hedw., *D. scoparium*, *Eurhynchium pulchellum* (Hedw.) Jenn., *H. splendens*, *Leucolepsis canthoneuron* (Schwaegr.) Lindb., *Neckera douglasii* Hook., *P. schreberi*, *Rhacomitrium lanuginosum* (Hedw.) Brid.	[153]
	*P. commune*	[119]
	*Rhytidiadelphus squarrosus* (Hedw.) Warnst.	[152]
	*Rhytidium rugosum* (Hedw.) Kindb., *Palustriella commutata* (Hedw.) Ochyra, *Homalothecium philippeanum* (Spruce) Schimp., *Anomodon attenuatus* (Hedw.) Ignatov & Fedosov, *H. splendens*	[127]
	*C. purpureus*	[150]
	*D. pulvinatus*, *S. crassipilum*, *O. anomalum*, *D. scoparium*, *P. juniperinum*, *P. piliferum*	[130]
	*G. pulvinata*, *T. subulata*, *W. controversa*, *L. sciuroides*, *H. cupressiforme*, *H. sericeum*, *N. complanata*, *M. undulatum*	[131]
**Fungi**		
*Cryptococcus neoformans*	*Scapania verrucosa* Heeg.	[154]
*Candida albicans*	*H. lutescens*, *H. cupressiforme*, *H. sericeum*, *T. delicatulum*, *H. nitens*, *C. molluscum*, *E. striatulum*	[134]
	*T. subulata*, *L. sciuroides*, *H. cupressiforme*, *H. sericeum*, *M. undulatum*	[131]
	*B. argenteum*	[138]
	*A. angusta*	[80]
	*P. lyellii*	[139]
*Aspergillus fumigatus*	*D. scoparium*	[141]
	*M. polymorpha*, *A. undulatum*, *P. patens*	[155]
	*S. verrucosa*	[154]
	*P. lyellii*	[139,156]

## Data Availability

No new data were created or analyzed in this study. Data sharing is not applicable to this article.

## Abbreviations

The following abbreviations are used in this manuscript:

AOX	alternative oxidase
APx	ascorbate peroxidase
BACs	bioactive compounds
C3	C3 patway or Calvin cycle in the photosynthesis
C4	C4 pathway or Hatch-Slack cycle in the photosynthesis
CAM	crassulacean acid metabolism
CAT	catalaze
CCD	carotenoid cleavage dioxygenases
CYP74	cytochrome family
DNA	deoxyribonucleic acid
EC	Enzyme Commission number
ENA-ATP	exitus Na-type adenosine triphosphatases
FA	fatty acid
FAAH	fatty acid amide hydrolase
FNS	flavon synthase
G+	Gramm positive
G−	Gramm negative
GA	gallic acid
GLVs	green leaf volatiles
GPx	glutathione peroxidase
GR	glutathione reductase
GSH	glutathione redused
GSSG	glutathione oxidized
GT/GST	glutathione transferase/glutathione S-transferase
HPL	hydroperoxide lyase
HPLC	high-performance liquid chromatography
KO	kaurene oxidase
MAX1 P450	family of cytochrome P450 enzymes
MPK	molecular pathway kinase
NR	nitrate reductase
PAL	phenylalanine ammonia lyase
PX	peroxidase
PUFAs	polyunsaturated fatty acids
QSAR	quantitative structure activity relationship
ROS	reactive oxygen species
RNS	reactive nitrogen species
SAR	structure activity relationship
SL	strigolactone
SM	secondary metabolite
SOD	superoxide dismutase
THC	tetrahydrocannabinol
TPS	terpene synthase
UV	ultraviolet
WHO	World Health Organization
